# Glial Derived Neurotrophic Factor and Schizophrenia Spectrum Disorders: A Scoping Review

**DOI:** 10.2174/011570159X340124241205095729

**Published:** 2024-12-13

**Authors:** Valerio Ricci, Domenico De Berardis, Giovanni Martinotti, Giuseppe Maina

**Affiliations:** 1 Ospedale San Luigi Gonzaga, Università degli Studi di Torino, Italia; Regione Gonzole, 10, Orbassano 10043, Torino, Italia;; 2 Dipartimento di Salute Mentale, Servizio Psichiatrico di Diagnosi e Cura, Ospedale “Giuseppe Mazzini”, Azienda Sanitaria Locale 4, 64100 Teramo, Italia;; 3 Dipartimento di Neuroscienze, Imaging e Scienze Cliniche, Università degli Studi Gabriele d’Annunzio Chieti-Pescara, 66100 Chieti, Italia;; 4 Dipartimento di Neuroscienze “Rita Levi Montalcini”, Università degli Studi di Torino, Torino, Italia

**Keywords:** GDNF, neurotrophic factors, schizophrenia, psychosis, cognitivity, substance-induced psychosis

## Abstract

**Background:**

Psychotic disorders, characterized by altered brain function, significantly impair reality perception. The neurodevelopmental hypothesis suggests these disorders originate from early brain development disruptions. Glial-derived neurotrophic factor (GDNF) is crucial for neuronal survival and differentiation, especially in dopaminergic neurons, and shows promise in neurodegenerative and neuropsychiatric conditions.

**Objectives:**

This scoping review aims to examine the role of GDNF in schizophrenia spectrum disorders and substance-induced psychoses, integrating knowledge on the neurobiological mechanisms and therapeutic potential of GDNF.

**Methods:**

A comprehensive literature search was conducted using PubMed and Scopus databases from January 2001 onwards. Data extraction focused on GDNF levels, cognitive function, antipsychotic treatment effects, and genetic studies.

**Results:**

The review included 25 studies (18 human, 7 animal). While some studies demonstrated inconsistent results regarding GDNF serum levels in schizophrenic patients, the majority reported correlations between GDNF levels and cognitive functions. Animal studies underscored GDNF's role in stress response, drug-induced neurotoxicity, and dopamine signaling abnormalities. Genetic studies revealed potential associations between GDNF gene polymorphisms and schizophrenia susceptibility, though findings were mixed.

**Discussion:**

GDNF plays a significant role in cognitive functions and neuroprotection in schizophrenia. The variability in study results underscores the complexity of GDNF's involvement. The therapeutic potential of GDNF in psychotic disorders remains unclear, necessitating further research to clarify its efficacy and safety.

**Conclusion:**

This review emphasizes the importance of integrated biomarker strategies, gene therapy approaches, and precision medicine in advancing the understanding and treatment of psychotic disorders.

## 1 INTRODUCTION

### 1.1 Psychotic Disorders

Psychotic disorders are marked by altered brain function, leading to significant impairments in the definition of reality. These disorders manifest through symptoms such as disorganized thought, including incoherence, derailment, tangentiality, disorganized behavior, or abnormal motor behaviors like catatonia. Delusions are fixed beliefs that remain unchanged despite contradictory evidence. Hallucinations involve false sensory experiences without external stimuli, with hearing voices being the most common. Negative symptoms encompass alogia, apathy, anhedonia, affective flattening, and avolition [1, 2].

According to the DSM-5, psychotic disorders fall under “Schizophrenia and other psychotic disorders,” including substance-induced psychotic disorders and those caused by medical conditions. In contrast, the ICD-11 classifies “Schizophrenia spectrum and other primary psychotic disorders” as a group including schizophrenia, schizoaffective disorder, acute and transient psychotic disorder, schizotypal disorder, delusional disorder, other primary psychotic disorders, and unspecified primary psychotic disorders. Non-primary psychotic disorders, like those due to medical conditions or substance use, are categorized separately [3].

The exact causes remain elusive. Psychotic disorders are known for their varied origins, with numerous theories proposed to explain their onset. The widely supported stress-vulnerability model suggests that disruptions in early brain development due to either genetic or environmental influences set the stage for vulnerability. Environmental risk factors for developing psychosis may include both biological elements, like infections, and psychological stressors, such as traumatic events or abuse in childhood [4, 5]. A prominent theory is the neurodevelopmental hypothesis. This theory emerged from analyses of cognitive, positive, and negative symptoms in schizophrenia patients alongside imaging studies. It posits that schizophrenia and other psychotic disorders originate during brain development, suggesting a fundamental disruption in the neurodevelopmental process [6]. During the initial phases of brain development, the typical progression of brain maturation and neuron formation can be adversely affected by certain pathological conditions. These conditions can intervene at various stages, including during cell growth, differentiation, the movement of cells to their intended locations, the refinement of neural connections through synaptic pruning, and the natural process of cell death [7, 8]. It is believed that such disturbances in the development of the nervous system, particularly in the transmission of signals and the formation of neuronal networks, can give rise to the symptoms associated with schizophrenia. The neurodevelopmental hypothesis posits that abnormalities in neuronal migration, the establishment of neuronal connections, and the adaptability of neural structures can result in significant changes in specific areas of the brain, pivotal for psychotic onset [9]. Neurotrophic factors, crucial for the nervous system's development and maintenance, support neuron survival and help shape neural connections, playing a significant role in the brain's adaptability to new information and in the pathology of schizophrenia. They are special proteins that play a key role in the growth, maintenance, and survival of certain types of nerve cells, including those that release dopamine, serotonin, acetylcholine, and glutamate. These factors help neurons in two main ways: They help decide which neurons survive and which die by sending signals inside and outside the cells. This process ensures that the brain retains the neurons it needs and eliminates those it does not. They help shape how neurons connect and communicate with each other, both when the brain is developing and after it is fully grown. This remodeling of connections is essential for learning, memory, and the brain's ability to adapt to new information.

### 1.2 Glial Derived Neurotrophic Factor (GDNF)

Glial-derived neurotrophic factor (GDNF) belongs to a novel family of neurotrophic factors related to the transforming growth factor-beta (TGF-β) superfamily. Isolated in 1993 from the rat glial cell line B49, GDNF enhances the survival and differentiation of embryonic midbrain dopaminergic neurons, increasing their high-affinity dopamine uptake [10]. This growth factor has generated optimism for new drug therapies for Parkinson’s disease [11-18]. GDNF is initially synthesized as an inactive preproGDNF of 211 amino acids, which is cleaved to yield the mature protein of 134 amino acids. It is N-glycosylated and contains seven cysteine residues, indicating a distant relationship with the TGF-β family [10-19]. GDNF has been classified as a member of the cysteine knot growth factor superfamily due to its structural similarity [20, 21]. The REarranged during Transfection (Ret) proto-oncogene has been identified as the signaling receptor for GDNF, requiring the accessory protein GDNF family receptor alpha 1 (GFRα1) for activation [22, 23]. GFRα1 binds to GDNF, forming a complex that interacts with Ret, leading to Ret dimerization and autophosphorylation. Other co-receptors, such as GFRα2, GFRα3, and GFRα4, have also been identified [24, 25]. *In vivo* studies corroborate GDNF's impact on dopaminergic neurons, showing its potential to enhance neurite length, cell dimensions, and dopamine absorption [26, 27]. However, knockout models for GDNF and its receptors indicate that GDNF is not essential for the embryonic development of dopaminergic neurons [28, 29]. Experimental findings demonstrate GDNF's efficacy in rescuing dopaminergic neurons in various Parkinson’s disease models, such as 1-Methyl-4-phenyl-1,2,3,6-tetrahydropyridine (MPTP)-induced degeneration [30] and 6-hydroxydopamine lesions [31, 32]. Given its efficacy, GDNF shows promise for treating motoneuron disorders and neurodegenerative conditions. GDNF, neurturine (NTN), and proliferation suppressor protein (PSP) enhance motor neuron survival *in vitro*, with evidence supporting their protective roles *in vivo* [33]. The ongoing exploration of GDNF's role in stress mechanisms further underscores its importance in neurobiological resilience [34, 35].

### 1.3 GDNF in Psychiatry

Several pieces of evidence suggest GDNF's potential utility in confronting neurodegenerative and neuropsychiatric conditions, such as Alzheimer's disease, Parkinson's disease, substance dependence, susceptibility to stress, and mood disorders [36-40]. Preclinical trials have shown that exposure to chronic unpredictable stress (CUS), a model for depression, results in depression-like behaviors and a reduction in hippocampal GDNF expression [41]. Remarkably, chronic administration of tricyclic antidepressants reverses these depression-like behaviors and normalizes hippocampal GDNF levels [42]. Investigations into GDNF's role in Major Depressive Disorder (MDD) pathophysiology have also been conducted in human subjects, revealing a significant decrease in serum, plasma, and messenger Ribonucleic Acid (mRNA) GDNF levels in MDD patients compared to healthy controls [43]. This finding is further supported by a recent meta-analysis assessing GDNF alterations in depressed patients [44], thus indicating a general trend towards reduced GDNF levels in MDD. Nonetheless, certain studies have reported increased GDNF levels in specific brain regions of MDD patients (Michel *et al*., 2008), such as a post-mortem study noting elevated GDNF in the parietal cortex of MDD subjects [45]. This variance may be attributed to the relatively small sample sizes of MDD patients (n =7) and healthy controls (n = 14) in these investigations. Additionally, GDNF's involvement has been observed in anxiety disorders [46], bipolar disorder [47], and obsessive disorders [48].

Regarding the role of GDNF in schizophrenia, studies often appear controversial. The occurrence of growth-related anomalies (such as low birth weight, delayed maturation, enlarged ventricles, and atrophy) frequently observed in schizophrenia cases supports the neurodevelopmental hypothesis [49, 50]. GDNF is a critical neurotrophic factor that facilitates the growth, differentiation, and survival of nerve cells during development and also maintains continuity and plasticity in adult neurons [51]. This neurotrophin is particularly significant in the mammalian brain, mainly for its role in supporting the survival of motor and dopaminergic neurons, suggesting its potential relevance to the neurodevelopmental hypothesis of schizophrenia [52]. Studies on GDNF levels in schizophrenia have yielded inconsistent results, where data indicate that serum GDNF levels correlate with cognitive symptoms [53, 54]. Emerging evidence suggests that dysfunctional GDNF and NGF signaling may contribute to structural brain alterations in schizophrenia patients, with serum NGF levels significantly correlated with reduced gray matter volume in specific brain regions [55]. Studies have shown that atypical antipsychotics, used in treating schizophrenia, increase NGF and GDNF levels, with patients treated with these drugs showing greater cognitive improvement than those treated with typical antipsychotics [56]. Shao *et al*. [57] examined the GDNF release from rat C6 glioma cells by antipsychotic drugs (clozapine, quetiapine, and haloperidol) and found that these drugs enhanced GDNF release.

Considering this information, the objective of this scoping review is to systematically examine the role of the Glial Cell Line-Derived Neurotrophic Factor (GDNF) in schizophrenia spectrum disorders, including schizophrenia, schizoaffective disorders, and delusional disorders. Additionally, we aim to broaden the scope by encompassing substance-induced psychoses, which currently hold significant clinical interest and represent a considerable proportion of psychosis cases. Furthermore, substances of abuse, particularly psychostimulants with high dopaminergic activity, are a critical area of study regarding neurotoxic brain damage and the subsequent reparative actions of GDNF. Our focus is on integrating existing knowledge to elucidate the neurobiological mechanisms and therapeutic potential of GDNF in these contexts.

## 2 METHODS

Following the methodology proposed by Levac *et al*. [57] and the guidelines from the Preferred Reporting Items for Systematic Reviews and Meta-Analyses extension for Scoping Reviews, a scoping review was executed. This process involved defining the research question, identifying relevant studies, selecting studies, charting data, and collating and reporting the findings. This review aimed to present a comprehensive overview of the literature without concentrating on a particular research topic [58].

### 2.1 Search Terms and Selection Criteria

A comprehensive literature search was conducted using the PubMed (*via* MEDLINE) and Scopus databases on December 21, 2023, covering the period from January 2001 onwards. Only original articles written in English were included. Eligible studies were experimental and observational, including post-marketing surveillance reports, case reports, and case series. Exclusion criteria included non-original research (*e.g*., reviews, commentaries, editorials, book chapters, and letters to the editor), non-full-text articles (*e.g*., meeting abstracts), and articles not in English. Research incorporating animal or *in vitro* experiments was also considered. While letters to the editor, conference proceedings, and book chapters were not directly included in the review, they were referenced for additional secondary sources. The search string used was: (GDNF OR Glial Derived Neurotrophic Factors OR Neurotrophic Factors) AND (psychosis OR schizophrenia OR schizoaffective OR substance-induced psychosis OR Cannabis psychosis OR Cocaine psychosis OR METH psychosis OR drug-induced psychosis). A total of 235 articles were identified (134 from PubMed, 101 from Scopus), and 25 were selected based on predefined criteria (18 human studies, 7 animal studies). The exclusion of the other 210 articles was due to irrelevance (170 articles) or non-compliance with selection criteria, including reviews, letters to the editor, commentaries, book chapters, non-English papers, and duplicates (40 articles). Findings were organized and presented according to study type in Tables **1** and **2**, providing a detailed and comprehensive view of the data consistent with the descriptive approach of the review.

### 2.2 Inclusion Criteria

Clinical diagnosis of schizophrenia spectrum disorders was made according to the DSM-5 criteria, including Substance/Medication-Induced Psychotic Disorder but excluding Psychotic Disorder Due to Another Medical Condition. Plasma or serum GDNF levels were assessed in human studies. For animal studies, both *in vivo* and *in vitro* studies were considered. Studies involving patients older than 18 years and up to 65 years were included. As for animal studies, both *in vivo* and *in vitro* research were included.

### 2.3 Exclusion Criteria

It was required that patients undergo comprehensive evaluations to rule out organic causes of psychosis. This included neuroimaging to exclude brain anomalies, electroencephalogram to exclude epilepsy, lumbar puncture to rule out encephalitis, neuropsychological assessments to evaluate for mental retardation, personality disorders, and dementia, and complete bloodwork to exclude infectious, hormonal, or vitamin-related conditions.

### 2.4 Data Extraction and Outcome Measures

The literature search and selection process were conducted by two investigators, Valerio Ricci (V.R.) and Domenico Deberardis (D.D.B.), under the guidance of supervisors Giuseppe Maina (G.M.) and Giovanni Martinotti (G.M.). This involved a two-stage independent review by the investigators, followed by a collaborative cross-check to ensure consistency and thoroughness.

**Fig. (1):**
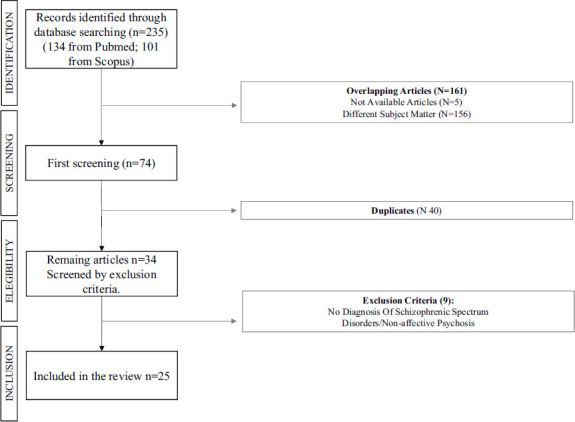
Flow-chart of study search and selection process.

The search terms and strategies adapted are reported in the Flow Chart (Fig. **1**). The following parameters were extracted from each eligible article: first author, publication year, language of the full text, diagnostic system, number of subjects, treatment used, and measurement of GDNF levels in plasma or serum, as well as brain tissue in mice (striatum, prelimbic cortex, ventral hippocampus, nucleus accumbens shell, and nucleus accumbens core). The primary outcome was serum or plasma GDNF levels in psychotic patients, the influence of antipsychotic treatment from baseline to post-treatment, the impact on cognitive function, and the role of the GDNF gene in psychosis. If a study reported GDNF concentrations for multiple time points within our predefined periods, we considered the data recorded at baseline and the last time point within the range for the overall effect analysis.

### 2.5 Data Charting Process and Analysis

Data collation focused on summarizing information from included studies. A narrative synthesis was undertaken through established guidance. A standard data extraction form was created, and the information was charted as follows: article title, first author’s name, year, country, study framework, study participants, features and strategies of the studies, measurement tools used, and main findings. Two reviewers separately extracted the data, and discrepancies in the data extraction were settled through consensus among the reviewing team.

## 3 RESULTS

### 3.1 Clinical and Genetic Findings in Human Studies

#### 3.1.1 GDNF and Cognitivity

Research on the relationship between GDNF and cognitive function in schizophrenia has progressed over the years, building on foundational studies to explore new dimensions and therapeutic implications.

Xiao [56] investigated the serum levels of BDNF and GDNF and their relation to cognitive function in first-episode, drug-naïve psychotic patients. The study presented evidence that levels of both neurotrophic factors were correlated with cognitive test performance. This suggested a significant involvement of BDNF and GDNF in the early stages of cognitive impairments associated with schizophrenia, highlighting their potential as biomarkers for cognitive dysfunction in this condition.

This observation was reinforced by a subsequent study by Niitsu and colleagues [18], which further explored serum levels of GDNF in schizophrenic patients treated with antipsychotics, comparing them with control subjects to assess clinical outcomes. Although no significant differences in GDNF levels were found between the two groups, a correlation was identified between GDNF levels and specific cognitive functions and symptoms. This finding underscores the complex relationship between neurotrophic factors and the cognitive aspects of schizophrenia. This study builds on the findings of Xiao [56], suggesting that GDNF may play a role in cognitive functions even in medicated patients.

Skibinska *et al*. [59] observed a temporal decrease in GDNF levels without significant differences between patients and controls. This study added a temporal dimension to the understanding of GDNF levels in schizophrenia, suggesting that GDNF levels may fluctuate over time in relation to disease progression.

Chu *et al*. [60] identified an inverse correlation between GDNF levels and clinical symptom severity and cognitivity in unmedicated patients. This finding aligned with previous observations, such as those by Skibinska *et al*. [59], highlighting a potential role of GDNF in modulating the severity of schizophrenia symptoms.

Several authors have correlated BDNF and GDNF levels with cognitive dysfunction. For instance, Tang *et al*. [61] investigated the differential roles of these neurotrophic factors in cognitive functioning within schizophrenia. They compared the levels of BDNF and GDNF between patients with deficit *versus* non-deficit schizophrenia and examined their correlation with cognitive abilities. Their study revealed that both patient groups exhibited reduced BDNF levels compared to controls, while higher levels of GDNF were associated with enhanced cognitive performance, particularly in patients with deficit schizophrenia. This study expanded on previous research by specifically examining subtypes of schizophrenia and their unique neurotrophic profiles. Similarly, Turkmen *et al*. [62] built on these findings by studying the relationship between cognitive functions and neurotrophic factors, including BDNF, GDNF, NGF, and Klotho, in individuals diagnosed with schizophrenia. Their findings highlighted a notable deficit in these neurotrophic factors among patients when compared to a control group. Interestingly, post-treatment observations indicated an elevation in BDNF levels, alongside a positive correlation between improvements in certain cognitive functions and the levels of BDNF, GDNF, and Klotho, specifically within the control group. This study reinforced the earlier findings of Tang *et al*. [61] and others, suggesting that neurotrophic factors play a crucial role in cognitive improvement following treatment.

#### 3.1.2 GDNF in Substance Misuse and the Impact of Antipsychotic Medications

Research on the role of GDNF in substance misuse and its interaction with antipsychotic medications has provided valuable insights into the complex interplay of neurotrophic factors in psychiatric conditions.

Tunka *et al*. [63] conducted an assessment across various psychiatric disorders, revealing uniformly lowered BDNF levels among all patient groups, with GDNF levels exhibiting variability. This indicates a broader, differential involvement of neurotrophic factors in psychiatric conditions, hinting at distinct glial functions between schizophrenia and mania. This study laid the groundwork for understanding the differential roles of neurotrophic factors in various psychiatric disorders.

Krivoy *et al*. [64] extended the investigation to treatment responses, examining vascular endothelial growth factor (VEGF), NGF, and GDNF levels in clozapine responders *versus* non-responders. They found no significant differences, which may indicate that these neurotrophic factors do not straightforwardly differentiate treatment efficacy in schizophrenia. This study built on the findings of Tunka *et al*. [63] by exploring the potential roles of multiple neurotrophic factors in treatment response.

Ye *et al*. [65] discovered significantly lower GDNF levels in patients with tardive dyskinesia, proposing GDNF's involvement in the pathogenesis of this condition. This finding suggested a specific role for GDNF in the development of side effects associated with antipsychotic treatment, complementing the broader findings of Krivoy.

Tikir *et al*. [66] investigated the role of neurotrophic factors GDNF and NGF in schizophrenia, comparing their levels in patients and healthy controls and examining their relationship with the Positive and Negative Syndrome Scale (PANSS) scores and the duration of untreated psychosis (DUP). The research included 45 schizophrenia patients who had not taken antipsychotics and 45 matched controls. Findings showed significantly lower GDNF and NGF levels in patients than in controls, with no correlation between DUP and these neurotrophic levels. However, a positive correlation existed between PANSS scores for general psychopathology, negative symptoms, and DUP. The study suggested that GDNF and NGF levels could be markers of schizophrenia onset and progression, emphasizing the importance of early treatment due to neurodevelopmental changes early in the disease. This study built on the earlier findings by providing a more detailed examination of the relationship between neurotrophic factors and clinical outcomes in schizophrenia.

Ermakov *et al*. [67] showed increased cytokines and neurotrophic factors, supporting the complexity of the inflammatory and neurotrophic landscape in psychosis. This study suggested a multifaceted neurobiological underpinning involving both neurotrophic and inflammatory pathways, providing a broader context for the findings of earlier studies [65, 66]. Considering therapies other than antipsychotic treatments, Akkus *et al*. [68] found no correlation between GDNF levels and electroconvulsive therapy in schizophrenic patients. This result contrasts with previous studies that suggested a relationship between neurotrophic factors and cognitive functions, highlighting the need for further research to clarify these discrepancies.

#### 3.1.3 GDNF and Genetic Studies in Psychosis

Research into the genetic underpinnings of GDNF and its role in psychosis has evolved over the years, highlighting the complexity of genetic contributions to the disorder.

The investigation into the genetic variations of the GDNF gene began with Lee *et al*. [9], who explored GDNF gene polymorphisms in schizophrenia patients. Their study did not uncover significant allelic differences, suggesting that not all genetic variations within the GDNF pathway may be directly involved in the pathogenesis of psychosis. This initial finding set the stage for more detailed genetic analyses in subsequent years.

Building on this early work, Michelato's study [69] on an Italian cohort examined the association between schizophrenia susceptibility and the GDNF gene's 3' untranslated region (Adenosine Guanine Guanine) and repeat polymorphism. Michelato identified a significant difference in allele frequencies between patients and controls, with protective alleles being more common in the control group. This finding introduced the possibility that specific GDNF gene variants could serve as protective factors against schizophrenia, adding a new layer of complexity to the genetic landscape of the disorder. Contrasting with these findings, Williams *et al*. [70] conducted a thorough analysis and found no substantial evidence to support the GDNF gene as a susceptibility gene for schizophrenia. This outcome mirrored the findings of Lee *et al*. [9] and suggested the complexity of genetic contributions to the disorder, emphasizing that the role of GDNF in schizophrenia may not be straightforward and could involve multiple genetic and environmental interactions.

Further advancing the field, Souza and colleagues [71] explored the relationship between polymorphisms in the GFRA (GDNF family receptor alpha) genes and schizophrenia, as well as the response to clozapine treatment. Their research spotlighted significant associations between specific variants in GFRA3 and GFRA1 with schizophrenia and identified a GFRA2 haplotype linked to improved clozapine response. This suggested a nuanced genetic influence on treatment efficacy. Additionally, Souza explored the genetic underpinnings of treatment side effects, investigating GFRA2 gene variations in susceptibility to tardive dyskinesia (TD), a common side effect of prolonged antipsychotic medication in schizophrenia. They found significant associations with TD risk, particularly for rs4739285 and rs4739217, highlighting the influence of specific allele combinations and age on TD susceptibility. These findings underscored the intricate interplay between genetic variations and both therapeutic outcomes and side effects in schizophrenia treatment [72]. Expanding the genetic inquiry further, MA and colleagues [39] included GDNF tag SNPs in their study, exploring their link to depression, heroin dependence, and psychosis within a Chinese population. Their findings pointed to genetic markers that elevate the risk for heroin dependence and depression, positing GDNF as a susceptibility gene for these conditions. This study broadened the scope of neurotrophic factors in psychiatric genetic research, indicating that the influence of GDNF extends beyond schizophrenia to other psychiatric conditions as well.

### 3.2 Animal Studies

In a series of pioneering studies, researchers have explored the complex interactions between neurotrophic factors, particularly glial cell line-derived neurotrophic factor (GDNF), and their influence on the pathophysiology of schizophrenia and related behavioral and molecular alterations. The narrative that unfolds from these investigations reveals both convergences and divergences in how alterations in GDNF expression and signaling pathways contribute to schizophrenia's etiology and symptomatology, as well as offering insights into potential therapeutic targets. Animal studies provide a broad perspective on the neurotoxic effects of psychostimulant substances in specific brain areas, offering important avenues for future research.

Buhusi *et al*. [73] investigated the impact of chronic stress on latent inhibition in mice with GDNF deficiencies. Their study illustrated the vulnerability of specific genetic backgrounds to environmental stressors, further complicating the schizophrenia narrative. This study emphasized the role of GDNF deficits in stress-induced pathological behaviors and underscored the importance of considering both genetic and environmental factors in understanding and treating psychiatric disorders.

Brown *et al*.'s investigation [74] into the effects of environmental conditions on nicotine sensitization and brain neuroplasticity in a rat model mimicking schizophrenia presents a nuanced view of how external factors can influence disease outcomes. By subjecting rats treated with neonatal quinpirole (NQ) to induce dopamine receptor sensitivity to different housing conditions-enriched environments *versus* isolation-Brown found that environmental enrichment did not alter nicotine sensitization in NQ rats but did reduce sensitization in control rats. Furthermore, environmental enrichment elevated GDNF levels in the nucleus accumbens of both NQ and nicotine-exposed control rats. This finding accentuates the profound impact of the environment on behavioral responses and brain plasticity, especially during adolescence, and highlights the potential of environmental interventions in moderating neuroplasticity and drug sensitization effects.

Casserly *et al*. [75] explored the effects of methamphetamine on GDNF expression and its receptors, revealing how acute and chronic exposure to the drug differently modulates GDNF levels and signaling pathways, thereby contributing to psychosis risk. Their findings highlight the delicate balance of neurotrophic signaling in the brain and its susceptibility to external influences, such as drug exposure, which may precipitate or exacerbate schizophrenia-like symptoms.

Gill *et al*. [76] added another layer to the story by demonstrating how NQ treatment amplifies the rewarding properties of nicotine and its effects on BDNF and GDNF, identifying the adenosine A2A receptor as a key player in mitigating these effects. This insight not only opens up new avenues for treating nicotine addiction but also suggests potential strategies for addressing comorbid nicotine addiction and schizophrenia.

Mätlikh and colleagues [77] stand out for their innovative approach to understanding the role of increased endogenous GDNF expression in the brain and its implications for dopamine signaling abnormalities akin to those observed in schizophrenia. By developing a novel *in vivo* method to conditionally increase GDNF expression without the negative impacts of ectopic overexpression, they uncovered significant molecular, cellular, and functional alterations in dopamine signaling within key brain areas. Their discovery points to the adenosine A2a receptor (A2AR) as a potential mediator through which GDNF may exert its influence on dopaminergic dysregulation, offering a new angle on therapeutic interventions.

Semba's research [78] delved into the effects of subchronic administration of phencyclidine (PCP), a compound notorious for inducing schizophrenia-like symptoms, on the GDNF system within the ventral midbrain-a pivotal region for dopamine neuron functionality. By treating male Wistar rats with PCP over a span of 10 days, Semba observed an uptick in GDNF and c-ret mRNA levels within the substantia nigra compacta (SNC) and ventral tegmental area (VTA). This increase suggests that PCP may disrupt the GDNF system's support of dopamine neurons, shedding light on a possible route through which PCP elicits schizophrenia-like symptoms and underlining the critical interplay between glutamatergic and dopaminergic systems in the disease's pathology.

Valvassori *et al*.'s [79] investigation into the effects of Haloperidol on ketamine-induced behavioral changes, neurotrophic factors, and epigenetic markers further enriches the narrative by illustrating the complex interplay between pharmacological interventions, neurotrophic factor levels, and epigenetic modifications in the context of schizophrenia. This study underscores the potential side effects and limitations of current treatments, pointing to the need for a deeper understanding of how these treatments interact with the brain's neurotrophic and epigenetic landscape.

## 4 DISCUSSION

The comprehensive review of the literature leads to several key conclusions:

### 4.1 GDNF In Humans Studies

#### 4.1.1 Serum Levels of GDNF in Psychotic Patients

Firstly, the studies on the serum levels of GDNF in schizophrenia yield inconsistent results. While some research suggests variations in GDNF serum levels among individuals with schizophrenia [53, 56, 60-65], others have not found significant differences [18-59]. Although a definitive link between GDNF gene expressions and schizophrenia remains to be established, there is suggestive evidence of a significant relationship with genes related to GDNF family receptors [72]. Moreover, Lee *et al*.'s study [9] on the GDNF gene polymorphisms in schizophrenia did not reveal significant allelic differences. This aligns with Williams *et al*.'s findings [70], which also found no significant evidence linking the GDNF gene to schizophrenia susceptibility, further emphasizing the intricate genetic landscape of the disorder. Michelato *et al*.'s [69] research suggests that certain GDNF gene variants may act as protective factors against schizophrenia, while Ma *et al*. [39] posits GDNF as a potential susceptibility gene for heroin dependence but not schizophrenia in the Chinese population.

#### 4.1.2 GDNF and Cognitive Support in Psychosis

A crucial understanding from our analysis, is the role of GDNF in cognitive functions. This aspect has received considerable attention in the field of neurology, especially concerning Parkinson's disease (PD) and Alzheimer's disease (AD). The theory of neurotrophic factor deficiency suggests these factors are vital for neuronal plasticity, learning, and other cognitive functions. Research indicates a correlation between cognitive decline in PD and the levels of neurotrophic factors in cerebrospinal fluid and plasma [80, 81]. GDNF, belonging to the transforming growth factor-β superfamily, has been central to our research for years, particularly its neuroprotective properties for dopaminergic neurons. Studies have demonstrated GDNF's ability to activate the PI3 kinase serine/threonine-specific protein kinase pathway, thereby increasing calbindin-D28Kilodalton (D28K) expression [82], reducing apoptosis in calbindin-D28K positive mouse neuroblastoma cell line 9D (MN9D) cells after 6-hydroxydopamine exposure [83], and promoting the survival of neurons in the substantia nigra pars compacta of rats also treated with 6-hydroxydopamine [84]. Furthermore, GDNF's importance in maintaining dopaminergic neurons in the nigrostriatal pathway has been documented [85]. Reduced peripheral serum GDNF levels observed in patients with mild cognitive impairment and Alzheimer’s disease suggest its potential role in these disorders [86]. Studies have also highlighted GDNF's influence on cognitive deficits in depression. Regarding schizophrenia, individuals exhibit a wide range of cognitive deficits, including in learning, memory, executive functions, and attention. Both animal models and clinical studies have identified a positive link between neurotrophic factors and cognitive impairments. GDNF, in particular, is essential for the survival of dopaminergic and motor neurons in the mammalian central nervous system (CNS). Research on GDNF heterozygous mutant mice, which showed reduced performance in water-maze learning tasks, underlines its critical role in cognitive functions. This evidence emphasizes GDNF's fundamental importance in sustaining cognitive abilities, further highlighting the necessity to explore neurotrophic factors for understanding and potentially treating cognitive impairments in schizophrenia. Indeed, these studies collectively indicate that high levels of GDNF correlate with cognitive performance, pointing to GDNF's therapeutic potential as a subject for future research. Relating the studies considered in our work all highlight a direct correlation between GDNF and cognitive performance in schizophrenic patients. This is a finding worthy of further investigation and future research [18, 56, 61, 62].

#### 4.1.3 GDNF and Substance-induced Psychosis

The studies collected thus far on the role of GDNF in substance abuse derive exclusively from animal studies [74-76, 79]. Extensive research has established that substance abuse, including psychostimulants, significantly impacts the normal operations and neurotransmitter signaling within the Central Nervous System (CNS). The exact processes by which they exert their effects remain somewhat unknown; however, it is recognized that they boost the levels of neurotransmitters such as dopamine, serotonin, and noradrenaline in the space outside cells by interacting with certain transporters. This interaction is associated with common outcomes like the development of tolerance, dependency, and the emergence of psychotic episodes. Furthermore, these substances are capable of causing damage to neural tissue through harmful mechanisms that are not yet fully understood, with ongoing studies attempting to elucidate these pathways. Neurotrophic factors play a crucial role in affecting specific groups of neurons, including those that produce dopamine, serotonin, and acetylcholine-targets that are similarly affected by psychoactive drugs. These neurotrophins impact neural function in two significant ways: they regulate the life and death of cells through sophisticated signaling both within and between cells, and they are involved in the reshaping of synaptic connections, which is essential for the growth and adaptation of neural networks throughout both the developmental phase and in mature individuals. In light of the studies we have examined, it seems that GDNF might play a crucial role in the onset of psychoses triggered by drugs like methamphetamine [79], Gill *et al*. [76], as well as being related to the neurobiological mechanisms of nicotine dependence in patients with schizophrenia [74].

### 4.2 GDNF in Animal Studies

From animal studies, the main findings identify the role of increased endogenous GDNF expression in the brain and its implications for dopamine signaling abnormalities similar to those observed in schizophrenia [77]. Additionally, the role of substances such as ketamine, methamphetamine, and phencyclidine [75, 78, 79] and the impact of drugs in altering dopaminergic transmission, including nicotinic receptors [74-76], are highlighted. The impact of chronic stress on latent inhibition in mice [73] with GDNF deficiencies illustrates the vulnerability of specific genetic backgrounds to environmental stressors, further complicating the narrative of schizophrenia. Chronic exposure to these substances persistently alters the structure and function of the hippocampus in rats, a region crucial for learning and memory processes [87].

## 5 LIMITATIONS

In examining this human data, it's crucial to recognize certain constraints that notably influence our interpretations and the subsequent conclusions we draw. Firstly, a significant limitation arises from the methodology employed in measuring levels of GDNF and other neurotrophins, which were assessed in serum rather than directly within the central nervous system (CNS). This distinction is critical because, although there is evidence to suggest that neurotrophins are capable of crossing the blood-brain barrier through a high-capacity, saturable transport mechanism [88, 89], the precise dynamics of this process and its efficiency in reflecting true CNS concentrations remain somewhat ambiguous. The lack of direct correlation studies between neurotrophin levels in the CNS and the peripheral system introduces a layer of uncertainty, hindering our ability to accurately map the neurotrophic factors' influence on neural health and disease directly from peripheral measurements.

The second limitation pertains to the scope of cognitive function assessment in the studies under review. The employment of a narrow selection of cognitive tests constrains the breadth of cognitive domains explored, offering insights into only specific areas of cognitive function. This approach presents a fragmented view of neurocognitive health and may overlook potential interactions between different cognitive domains or the broader impact of neurotrophins on overall cognitive performance. It underscores the imperative need for future research to not only incorporate a broader and more diverse set of cognitive assessments but also to utilize the findings from existing tests as a foundation for exploring neurocognitive functions across a more extensive spectrum of areas.

Finally, the third limitation is the limited number of studies available to us. This scarcity significantly impacts the robustness of our conclusions and the generalizability of our findings. The field's knowledge base is constrained not only by the number of studies but also by their diversity in design, populations studied, and neurotrophic factors analyzed. This limitation emphasizes the urgent need for more comprehensive research efforts. The current paucity of studies underscores the necessity for increased research activity aimed at exploring the multifaceted relationships between GDNF and schizophrenia across diverse populations and with a variety of methodological approaches.

In addition to these methodological constraints, it is crucial to address the conceptual complexities surrounding schizophrenia itself. Psychosis and schizophrenia-related disorders are often described as a “pseudo-genetic” disorder, where genetic predispositions alone do not fully account for its manifestation. There is an ongoing debate about the extent to which environmental factors and gene-environment interactions contribute to the development of schizophrenia. Studies suggest that what is often diagnosed as schizophrenia could encompass a range of conditions, sometimes referred to as pseudo-schizophrenia or schizophrenia-like psychosis, which mimic the symptoms but have different etiologies [90, 91]. This recognition prompts a call for more nuanced and comprehensive gene-environment studies [92]. Furthermore, psychiatry, from an epistemological standpoint, is still grappling with its scientific foundations, often compared to the dark ages of science. This critique highlights the need for a more profound understanding of schizophrenia, not just as a genetic or purely neurochemical disorder, but as a complex, multi-faceted condition that demands a holistic approach to research and treatment [90-93].

By acknowledging these broader considerations, we can better appreciate the challenges inherent in studying neurotrophic factors in the context of schizophrenia and strive towards more integrated and interdisciplinary research methodologies.

Another important aspect to consider is the role of neurotrophins in substance-induced psychoses, which remains uncertain. The studies presented here were conducted on animals, and the findings are controversial and do not form a definitive framework. However, these studies offer valuable insights into the neurotoxic effects of substances of abuse, making them particularly relevant for potential future research in humans. It is well-established that the use of psychostimulants can lead to lasting changes in brain structures. In humans, brain imaging studies have demonstrated that prolonged use of psychostimulants affects neuronal and axonal integrity in the dorsolateral prefrontal cortex [94]. Recent research indicates that chronic consumption of psychostimulants disrupts neuronal and axonal integrity, as evidenced by N-acetyl aspartate magnetic resonance spectroscopy in the dorsolateral prefrontal cortex [95], and is associated with reduced volume in specific brain areas such as the parahippocampal gyrus and parietal lobe [96]. These findings suggest that long-term exposure to psychostimulants may impair neural pathways and potentially be associated with neurotoxic cascades, linking to the hypothesis of a possible alteration in neurodevelopment in the genesis of schizophrenia and related disorders.

Recent studies have indicated that psychostimulants alter neuronal function and neurotransmission by competing with monoamine transporters such as DAT and VMAT-2. These transporters are involved in regulating intracerebral dopamine distribution. Chronic stimulation with psychostimulants enhances the activity of mesocorticolimbic dopaminergic neurons projecting from the ventral tegmental area (VTA) to the nucleus accumbens (NAc) and medial prefrontal cortex (mPFC), as well as the activity of glutamate neurons projecting from the mPFC to the VTA and NAc. Adaptations in the dopaminergic and glutamatergic systems following exposure to psychostimulants are significantly implicated in their neurotoxic effects and the development of psychosis. Excess dopamine and its analogues in neuronal circuits can damage neurons and facilitate cell death, as demonstrated by numerous *in vivo* and *in vitro* studies [97].

In this context, glial cell line-derived neurotrophic factor (GDNF) plays a neuroprotective role by promoting dopaminergic neuronal growth and maintaining synaptic plasticity. Consequently, damage caused by psychoactive substances may not only be direct but also due to the disruption of these factors and the loss of neuronal protection. Dopaminergic neurons in the substantia nigra and striatum express the TrkB receptor on their surface, and there is a high density of neurotrophic factors in that area [98, 99]. Experimental evidence has shown that GDNF and brain-derived neurotrophic factor (BDNF) prevent the spontaneous death of dopaminergic neurons in cultures of primary rat mesencephalic cells and protect these neurons from neurotoxic damage caused by exposure to MPP (1-methyl-4-phenylpyridinium) toxin [100, 101]. Therefore, it can be postulated that neurotrophic factors active on the dopaminergic pathway, such as GDNF and BDNF, support the survival and repair of mesencephalic dopaminergic neurons under neurotoxic conditions, at least in some experimental settings. Conclusively, the hypothesis of a neurotrophic alteration in the genesis of schizophrenia remains worthy of further investigation.

## CONCLUSION

Our study presents novel insights into the role of the Glial-Derived Neurotrophic Factor (GDNF) in schizophrenia spectrum disorders, offering a deeper understanding of its potential impact. In particular, this research enriches the literature on the neurodevelopmental hypothesis of schizophrenia, suggesting that disruptions in GDNF signaling may significantly contribute to the onset and progression of psychotic disorders. This scoping review also aims to expand the current knowledge on the role of GDNF in substance-induced psychoses, in addition to the more extensively studied brain-derived neurotrophic factor (BDNF) and nerve growth factor (NGF). Despite the mixed results, the involvement of GDNF cannot be overlooked, given the neurotoxic damage in dopaminergic areas induced by psychostimulants. Given the high prevalence of substance abuse among individuals with psychotic disorders, this approach is crucial. Our review explores the relationship between GDNF and cognitive functions in schizophrenia, providing insights into how GDNF levels correlate with cognitive impairments and its potential as a biomarker for cognitive dysfunction. We also discuss how variations in GDNF levels may influence the response to antipsychotic treatments and the development of treatment-related side effects, such as tardive dyskinesia. Furthermore, we investigate the genetic underpinnings of GDNF and its receptors, discussing how polymorphisms in these genes may affect susceptibility to schizophrenia and treatment outcomes.

Regarding the therapeutic aspect, although some evidence has explored the role of GDNF in alcohol use disorders in animal studies [102, 103] and in Parkinson's disease [23, 104], significant challenges remain in fully understanding GDNF's role in psychotic disorders. Current evidence does not yet conclusively support the use of GDNF in treating these disorders, highlighting the need for further research to substantiate these preliminary findings and clarify its therapeutic potential.

Our study aims to contribute to the exploration of GDNF's role in psychotic disorders and to encourage further investigation to validate these hypotheses. Future research should focus on developing integrated biomarker strategies that combine neurotrophic, genetic, and cognitive assessments to improve diagnostic and prognostic precision. Investigating gene-therapy approaches targeting GDNF expression, along with pharmacological methods to modulate its signaling pathway, is crucial to determine their true efficacy and safety as potential neuroprotective interventions for psychotic disorders. Additionally, understanding the impact of environmental factors on neuroplasticity and GDNF levels is essential for evaluating the potential of lifestyle and environmental interventions in treatment plans. Addressing addiction and comorbidities within psychotic disorder treatment frameworks remains a critical area for further investigation, particularly in utilizing GDNF's role in addiction pathways to manage concurrent substance use disorders. Future studies should adopt a precision medicine approach to identify psychotic disorder subgroups based on neurotrophic factor profiles and genetic markers, facilitating tailored interventions to meet individual patient needs. Longitudinal and developmental research is imperative to map the trajectory of GDNF levels and their impact on the disease course, providing insights into optimal intervention timings and strategies.

## AUTHORS’ CONTRIBUTIONS

The authors confirm their contribution to the paper as follows: V.R., D.D.B., Giovanni Martinotti (G.M.)., and Giuseppe Maina (G.M.) contributed to the research design and implementation, as well as the data analysis and manuscript writing. All authors reviewed the results and approved the final version of the manuscript.

## Figures and Tables

**Table 1:** Studies on GDNF in human samples.

**Authors**	**Year of Publication**	**Number of Patients**	**Results**	**Type of Study**
Lee *et al*.	2001	99	Not all genetic variations within the GDNF pathway may be directly involved in schizophrenia's pathogenesis.	Polymorphism screening
Michelato *et al*.	2004	200	Schizophrenia susceptibility and the GDNF gene's 3' UTR (AGG) in repeat polymorphism with protective alleles being more common in the control group.	Genetic association
Williams *et al*.	2007	673	No substantial evidence to support the GDNF gene as a susceptibility gene for schizophrenia.	Genetic association
Ma *et al*.	2013	384	GDNF as a susceptibility gene for heroin dependence and depression.	Genetic association
Niitsu *et al*.	2014	63	No significant differences in GDNF levels between 63 schizophrenic and 52 healthy controls. Correlation was identified between GDNF levels and cognitive functions and symptoms.	Serum level analysis
Tunka *et al*.	2015	33	GDNF levels were significantly lower in 33 schizophrenic patients compared to healthy controls.	Serum level analysis
Xiao *et al*.	2016	138	Increase in GDNF levels among 138 schizophrenic patients after atypical antipsychotic treatment.	Treatment response
Akkus *et al*.	2017	20	No correlation between GDNF levels and electroconvulsive therapy in 20 schizophrenic patients.	Treatment response
Skibinska *et al*.	2017	55	Temporal decrease in GDNF levels without significant differences between 55 schizophrenic patients and controls.	Serum level analysis
Xiao *et al*.	2017	58	Low peripheral levels in 58 patients experiencing FEP compared to healthy controls; levels are also negatively influenced by cannabis use.	Cognitive correlation
Krivoy *et al*.	2018	89	No significant differences in VEGF NGF and GDNF levels in clozapine responders *versus* non-responders in 89 schizophrenic patients.	Treatment response
Ye *et al*.	2018	75	Lower GDNF levels in 75 schizophrenic patients with tardive dyskinesia.	Serum level analysis
Souza *et al*.	2018	140	Associations between specific variants in GDNF family receptors linked to improved clozapine response in 140 schizophrenic patients.	Polymorphism screening
Chu *et al*.	2018	30	Inverse correlation between GDNF levels and symptom severity in 30 unmedicated schizophrenic patients.	Serum level analysis
Tang *et al*.	2019	58	BDNF and GDNF in schizophrenia and correlation with cognitive abilities. Patients from both groups exhibited reduced BDNF levels in contrast to controls whereas higher levels of GDNF were associated with enhanced cognitive performance.	Cognitive correlation
Tikir *et al*.	2021	45	Significantly lower GDNF and NGF levels in 45 schizophrenic patients with no correlation between DUP and neurotrophic levels.	Serum level analysis
Turkmen *et al*.	2021	85	Relationship between cognitive functions GDNF and Klotho. GDNF serum levels in 85 schizophrenic patients with cognitive disfunctions compared to control group.	Cognitive correlation
Ermakov *et al*.	2023	54	Increased inflammatory cytokines and neurotrophic factors (GDNF) in 54 schizophrenic patients.	Inflammatory markers

**Abbreviations**: BDNF: Brain-Derived Neurotrophic Factor GDNF: Glial Cell Line-Derived Neurotrophic Factor FEP: First-Episode Psychosis VEGF: Vascular Endothelial Growth Factor NGF: Nerve Growth Factor UTR: Untranslated Region DUP: Duration of Untreated Psychosis.

**Table 2:** Studies on GDNF in animal samples.

**Authors**	**Year of Publication**	**Results**	**Type of Study**
Semba *et al*.	2004	Uptick in GDNF and c-ret mRNA levels within the substantia nigra compacta (SNC) and ventral tegmental area (VTA) after treating male Wistar rats with phencyclidine.	Gene expression analysis
Buhusi *et al*.	2017	Impact of chronic stress on latent inhibition in mice with GDNF deficiencies illustrates the vulnerability of specific genetic backgrounds to environmental stressors.	Behavioral study
Brown *et al*.	2018	Environmental enrichment elevated GDNF levels in the nucleus accumbens of both neonatal quinpirole and nicotine-exposed control rats.	Environmental influence study
Gill *et al*.	2020	How NQ treatment amplifies the rewarding properties of nicotine and its effects on BDNF and GDNF identifying the adenosine A2A receptor as a key player in mitigating these effects.	Pharmacological study
Mätlikh *et al*.	2022	A2a receptor (A2AR) as a potential mediator through which GDNF may exert its influence on dopaminergic dysregulation within key brain areas.	Receptor interaction study
Casserly *et al*.	2023	Acute and chronic exposure to methamphetamine differently modulates GDNF levels and signaling pathways thereby contributing to psychosis risk.	Drug exposure study
Valvassori *et al*.	2023	How Haloperidol interacts with the brain's neurotrophic factor GDNF and epigenetic landscape.	Pharmacological interaction study

**Abbreviations**: A2A: Adenosine A2A Receptor; GDNF: Glial Cell Line-Derived Neurotrophic Factor; NQ: nicotine; mRNA: Messenger RNA.

## LIST OF ABBREVIATIONS

AD: Alzheimer's Disease
BDNF: Brain-derived Neurotrophic Factor
CNS: Central Nervous System
CUS: Chronic Unpredictable Stress
GDNF: Glial-derived Neurotrophic Factor
MDD: Major Depressive Disorder
mPFC: Medial Prefrontal Cortex
mRNA: Messenger Ribonucleic Acid
NQ: Neonatal Quinpirole
NAc: Nucleus Accumbens
PD: Parkinson's Disease
PSP: Proliferation Suppressor Protein
SNC: Substantia Nigra Compacta
TGF-β: Transforming Growth Factor-beta
VEGF: Vascular Endothelial Growth Factor
VTA: Ventral Tegmental Area

## STANDARDS OF REPORTING

PRISMA guidelines and methodology were followed.

## SUPPLEMENTARY MATERIAL

PRISMA checklist is available as supplementary material on the publisher’s website along with the published article.
