# Supplementary material for: Glial Derived Neurotrophic Factor and Schizophrenia Spectrum Disorders: A Scoping Review
Source: Curr Neuropharmacol. 2024 Dec 13;23(5):564–78. doi: 10.2174/011570159X340124241205095729 (PMC12163499; doi:10.2174/011570159X340124241205095729)
Supplement: Supplementary file 1 — PRISMA checklist is available as supplementary material on the publisher’s website along with the published article. [file CN-23-5-564_SD1.pdf]

Supplementary Material

Glial Derived Neurotrophic Factor and Schizophrenia Spectrum Disorders: A Scoping Review

Valerio Ricci[1,*], Domenico De Berardis[2], Giovanni Martinotti[3] and Giuseppe Maina[1,4]

[1]Ospedale San Luigi Gonzaga, Università degli Studi di Torino, Italia; Regione Gonzole, 10, Orbassano 10043, Torino, Italia; [2]Dipartimento di Salute Mentale, Servizio Psichiatrico di Diagnosi e Cura, Ospedale "Giuseppe Mazzini", Azienda Sanitaria Locale 4, 64100 Teramo, Italia; [3]Dipartimento di Neuroscienze, Imaging e Scienze Cliniche, Università degli Studi Gabriele d'Annunzio Chieti-Pescara, 66100 Chieti, Italia; [4]Dipartimento di Neuroscienze "Rita Levi Montalcini", Università degli Studi di Torino, Torino, Italia

1875-6190/25 $65.00+.00 **© 2025 Bentham Science Publishers**

PRISMA 2009 Checklist

Section/topic	#	Checklist item	Reported on page #
**TITLE**			
Title	1	**Identify the report as a systematic review, meta-analysis, or both.**<br>The title of the document clearly identifies it as a scoping review ("Glial derived neurotrophic factor and schizophrenia spectrum disorders: a scoping review") and focuses on examining the role of glial cell line-derived neurotrophic factor (GDNF) in schizophrenia spectrum disorders.	1
**ABSTRACT**			
Structured summary	2	**Provide a structured summary including, as applicable: background; objectives; data sources; study eligibility criteria, participants, and interventions; study appraisal and synthesis methods; results; limitations; conclusions and implications of key findings; systematic review registration number.**<br>The document includes a structured abstract that covers elements such as background, objectives, data sources, eligibility criteria, study appraisal and synthesis, results, limitations, conclusions, and implications. Therefore, it fulfills the PRISMA guideline requirements for a structured summary.	2
**INTRODUCTION**			
Rationale	3	**Describe the rationale for the review in the context of what is already known.**<br>The introduction of the document provides a clear rationale for the review, explaining the importance of studying glial cell line-derived neurotrophic factor (GDNF) in the context of schizophrenia and related psychotic disorders. It outlines existing knowledge about the neurodevelopmental hypothesis and the role of GDNF in neuropsychiatric conditions, setting a foundation for the study's objective.	4-6
Objectives	4	**Provide an explicit statement of questions being addressed with reference to participants, interventions, comparisons, outcomes, and study design (PICOS).**<br>The document specifies its objective as examining the role of GDNF in schizophrenia spectrum disorders, including substance-induced psychoses. It focuses on neurobiological mechanisms and the therapeutic potential of GDNF. Although the PICOS elements are not explicitly formatted, the objective covers participants (individuals with schizophrenia or substance-induced psychosis), outcomes (GDNF levels, cognitive function, and therapeutic implications), and study design (a scoping review).	6
**METHODS**			
Protocol and registration	5	**Indicate if a review protocol exists, if and where it can be accessed (e.g., Web address), and, if available, provide registration information including registration number.**<br>The document mentions following the guidelines from the PRISMA extension for Scoping Reviews and references methodology by Levac et al., but there is no indication of a registered protocol or specific registration information for this review. Thus, this checklist item appears unaddressed in terms of registration.	7
Eligibility criteria	6	**Specify study characteristics (e.g., PICOS, length of follow-up) and report characteristics (e.g., years considered, language, publication status) used as criteria for eligibility, giving rationale.**<br>The document defines clear eligibility criteria, including the study characteristics (experimental and observational studies involving human subjects aged 18 to 65, animal studies, and specific measures like GDNF levels) and report characteristics (original articles in English, excluding non-original research). It also details exclusion criteria, such as articles not meeting full-text or language requirements and studies unrelated to GDNF's role in psychosis, with explanations provided for each criterio.	7
Information sources	7	**Describe all information sources (e.g., databases with dates of coverage, contact with study authors to identify additional studies) in the search and date last searched.**<br>The document describes the information sources used for the literature search, specifying PubMed (via MEDLINE) and Scopus databases. It also provides the date of the last search, conducted on December 21, 2023. This aligns with the PRISMA requirement to document information sources and search date.	7
Search	8	**Present full electronic search strategy for at least one database, including any limits used, such that it could be repeated.**<br>The document includes a detailed search strategy, presenting the search terms and specific keywords used (e.g., "GDNF OR Glial Derived Neurotrophic Factors OR Neurotrophic Factors" and terms related to psychosis and schizophrenia). This allows for reproducibility of the search in the specified databases, meeting the PRISMA requirement for transparency in search strategy.	7
Study selection	9	**State the process for selecting studies (i.e., screening, eligibility, included in systematic review, and, if applicable, included in the meta-analysis).**<br>The document outlines the study selection process, which includes an initial screening and eligibility assessment. Two investigators independently reviewed the studies, followed by a cross-check to ensure consistency. This approach, involving multiple stages and independent review, satisfies the PRISMA requirement for describing the study selection process.	7
Data collection process	10	**Describe method of data extraction from reports (e.g., piloted forms, independently, in duplicate) and any processes for obtaining and confirming data from investigators.**<br>The data collection process is described in detail. It involved a two-stage independent review by the investigators, followed by collaborative cross-checking to ensure thoroughness and consistency. Data were extracted using a standard form that included study details like the article title, year, diagnostic	8

Section/topic	#	Checklist item	Reported on page #
		criteria, and primary outcomes, fulfilling the PRISMA guideline for transparency in data collection.	
Data items	11	**List and define all variables for which data were sought (e.g., PICOS, funding sources) and any assumptions and simplifications made.** The document specifies the data items extracted, including study author, publication year, language, diagnostic criteria, number of subjects, treatment used, and GDNF measurements in serum, plasma, or brain tissue. It also lists primary outcomes such as GDNF levels, cognitive function, and the influence of antipsychotic treatment, which aligns with the PRISMA guideline to define the key data items sought.	8
Risk of bias in individual studies	12	**Describe methods used for assessing risk of bias of individual studies (including specification of whether this was done at the study or outcome level), and how this information is to be used in any data synthesis**. The review employed a structured approach to assess the risk of bias in each included study. The Cochrane Risk of Bias Tool was utilized for randomized studies, while the Newcastle-Ottawa Scale (NOS) was applied to observational studies. Each study was independently evaluated by two reviewers, and discrepancies were resolved through consensus or consultation with a third reviewer when necessary. Bias was assessed at both the study and outcome levels, allowing us to account for potential bias in the synthesis and interpretation of findings. Studies identified with a high risk of bias were included in the review but marked as potentially less reliable in the narrative synthesis and discussion.	8-9
Summary measures	13	**State the principal summary measures (e.g., risk ratio, difference in means).** The document specifies that the primary summary measures included changes in GDNF levels, correlations with cognitive function, and associations with antipsychotic treatment outcomes. These measures provide a quantitative basis for evaluating the effects of GDNF on schizophrenia spectrum disorders, fulfilling the PRISMA requirement for defining principal summary measures.	8-9
Synthesis of results	14	**Describe the methods of handling data and combining results of studies, if done, including measures of consistency (e.g., I^2) for each meta-analysis.** The review describes a narrative synthesis approach to summarize findings from the included studies. Although no meta-analysis was conducted, the synthesis categorizes results by study type (human vs. animal studies) and outcome (GDNF levels, cognitive function, etc.), providing a clear structure for combining results. Consistency across studies was noted qualitatively, as required by PRISMA when statistical synthesis is not.	8-9
**Section/topic**	**#**	**Checklist item**	**Reported on page #**
Risk of bias across studies	15	**Specify any assessment of risk of bias that may affect the cumulative evidence (e.g., publication bias, selective reporting within studies).** To assess risk of bias across studies, the review considered potential publication bias by examining the range of sources included and assessing the consistency of reported outcomes. Any indication of selective reporting, such as the omission of negative findings in studies of GDNF's effects, was noted in the synthesis. Studies with high risk of bias were flagged to inform the interpretation of cumulative evidence, aligning with PRISMA's recommendation for evaluating bias across studies.	8-9
Additional analyses	16	**Describe methods of additional analyses (e.g., sensitivity or subgroup analyses, meta-regression), if done, indicating which were pre-specified.** The review included subgroup analyses to examine GDNF levels across different diagnostic groups within the schizophrenia spectrum and between human and animal studies. Although no formal sensitivity analysis was conducted, the review discusses the influence of variables such as study design and patient characteristics on the observed outcomes. This approach fulfills PRISMA's recommendation to detail additional analyses.	8
**RESULTS**			
Study selection	17	**Give numbers of studies screened, assessed for eligibility, and included in the review, with reasons for exclusions at each stage, ideally with a flow diagram.** The document provides a detailed account of the study selection process, including the total number of studies identified (235), those screened, assessed for eligibility, and the final 25 studies included. It mentions reasons for exclusion, such as irrelevance or non-compliance with eligibility criteria. Although no flow diagram is included, the text-based summary effectively describes the selection process and aligns with PRISMA's requirements	9
Study characteristics	18	**For each study, present characteristics for which data were extracted (e.g., study size, PICOS, follow-up period) and provide the citations.** The document outlines the characteristics of each study, including sample size, diagnostic criteria, treatments used, and GDNF measurement methods. It also categorizes studies by human vs. animal research, detailing key data points like GDNF levels, cognitive impacts, and antipsychotic treatment effects, meeting the PRISMA guideline for reporting study characteristics.	9
Risk of bias within studies	19	**Present data on risk of bias of each study and, if available, any outcome level assessment (see item 12).** The document includes a risk of bias assessment for each study, using structured tools such as the Cochrane Risk of Bias Tool and the Newcastle-Ottawa Scale, depending on the study design. Each study's bias level was reviewed by two independent assessors, with any high-bias studies flagged to inform	9-10-11

Section/topic	#	Checklist item	Reported on page #
		interpretations. While no outcome-level bias assessment is specifically mentioned, study-level bias is noted throughout, in alignment with PRISMA's guidelines.	
Results of individual studies	20	**For all outcomes considered (benefits or harms), present, for each study: (a) simple summary data for each intervention group (b) effect estimates and confidence intervals, ideally with a forest plot.** The review provides a narrative summary of the results for each included study, highlighting main outcomes such as GDNF levels and cognitive function associations. While effect estimates and confidence intervals are not reported due to the narrative synthesis format, key findings from individual studies are presented comprehensively. This approach meets the PRISMA recommendation for summarizing individual study results in a non-meta-analytical review.	9-14
Synthesis of results	21	**Present results of each meta-analysis done, including confidence intervals and measures of consistency.** As this review uses a narrative synthesis rather than a meta-analysis, no pooled effect estimates, confidence intervals, or quantitative measures of consistency (e.g., I^2) are provided. Instead, results are grouped by study type and outcome, with consistency discussed qualitatively where applicable. This approach aligns with PRISMA's recommendations for narrative synthesis in cases where meta-analysis is not feasible.	9-114
Risk of bias across studies	22	**Present results of any assessment of risk of bias across studies (see Item 15).** The document discusses potential publication bias and selective reporting across studies, particularly noting any indications of bias that could impact the cumulative evidence. Studies identified as high risk were highlighted in the discussion to inform the interpretation of findings and address the potential influence of bias on overall conclusions, meeting PRISMA's requirements for addressing cross-study bias.	9-14
Additional analysis	23	**Give results of additional analyses, if done (e.g., sensitivity or subgroup analyses, meta-regression [see Item 16]).** The review includes subgroup analyses to explore variations in GDNF levels and cognitive outcomes across different diagnostic subgroups within the schizophrenia spectrum and between human and animal studies. Although a formal sensitivity analysis was not performed, the document discusses how different study designs and participant characteristics may impact findings, addressing additional analysis requirements as recommended by PRISMA.	9-14
**DISCUSSION**			
Summary of evidence	24	**Summarize the main findings including the strength of evidence for each main outcome; consider their relevance to key groups (e.g., healthcare providers, users, and policy makers).** The review provides a detailed summary of the main findings, emphasizing the role of GDNF in cognitive function, neuroprotection, and potential therapeutic applications within schizophrenia spectrum disorders. It discusses the strength and consistency of evidence across studies, highlighting areas with promising findings and those requiring further research. This aligns with PRISMA's guidance to provide a comprehensive summary of evidence and its implications.	15-116
Limitations	25	**Discuss limitations at study and outcome level (e.g., risk of bias), and at review-level (e.g., incomplete retrieval of identified research, reporting bias).** The document discusses several limitations, including methodological constraints such as the use of serum rather than central nervous system measurements of GDNF, limited cognitive assessment tools, and a small number of available studies. It also acknowledges potential reporting bias and publication bias as limitations in the review's overall conclusions, meeting PRISMA's requirements for addressing limitations at multiple levels.	17-20
Conclusions	26	**Provide a general interpretation of the results in the context of other evidence, and implications for future research.** The review concludes by interpreting its findings within the broader context of schizophrenia research, emphasizing GDNF's potential role in cognitive and therapeutic outcomes. It suggests future research directions, such as exploring genetic and biomarker-based studies and developing more robust methods to assess GDNF's role in neuropsychiatric disorders. This response satisfies PRISMA's guidance for drawing conclusions and suggesting implications for future work.	20-21
**FUNDING**			
Funding	27	**Describe sources of funding for the systematic review and other support (e.g., supply of data); role of funders for the systematic review.** The document states that there was no specific funding provided for this review, and all authors confirmed no financial support influenced the review process or its conclusions. This meets PRISMA's requirement to disclose funding sources and the role of funders, ensuring transparency in the research process The review includes a clear statement on conflicts of interest, declaring that the primary authors (V.R., D.D.B., G.M., and G.M.) had no conflicting interests related to this work. This satisfies PRISMA's guideline to disclose any potential conflicts that might influence the review, supporting the credibility and transparency of the findings.	22

From: Moher D, Liberati A, Tetzlaff J, Altman DG, The PRISMA Group (2009). Preferred Reporting Items for Systematic Reviews and Meta-Analyses: The PRISMA Statement. PLoS Med 6(7): e1000097. doi:10.1371/journal.pmed1000097

For more information, visit: **www.prisma-statement.org**.
